# Recovery of Donor Hematopoiesis after Graft Failure and Second Hematopoietic Stem Cell Transplantation with Intraosseous Administration of Mesenchymal Stromal Cells

**DOI:** 10.1155/2018/6495018

**Published:** 2018-04-10

**Authors:** Nataliya Petinati, Nina Drize, Natalia Sats, Natalya Risinskaya, Andrey Sudarikov, Michail Drokov, Daria Dubniak, Alina Kraizman, Maria Nareyko, Natalia Popova, Maya Firsova, Larisa Kuzmina, Elena Parovichnikova, Valeriy Savchenko

**Affiliations:** ^1^Bone Marrow Transplantation Department, National Research Center for Hematology, Noviy Zikovsky pr, 4, Moscow 125167, Russia; ^2^Laboratory for Physiology of Hematopoiesis, National Research Center for Hematology, Noviy Zikovsky pr, 4, Moscow 125167, Russia; ^3^Laboratory for Molecular Hematology, National Research Center for Hematology, Noviy Zikovsky pr, 4, Moscow 125167, Russia

## Abstract

Multipotent mesenchymal stromal cells (MSCs) participate in the formation of bone marrow niches for hematopoietic stem cells. Donor MSCs can serve as a source of recovery for niches in patients with graft failure (GF) after allogeneic bone marrow (BM) transplantation. Since only few MSCs reach the BM after intravenous injection, MSCs were implanted into the iliac spine. For 8 patients with GF after allo-BMT, another hematopoietic stem cell transplantation with simultaneous implantation of MSCs from their respective donors into cancellous bone was performed. BM was aspirated from the iliac crest of these patients at 1-2, 4-5, and 9 months after the intraosseous injection of donor MSCs. Patients' MSCs were cultivated, and chimerism was determined. In 6 out of 8 patients, donor hematopoiesis was restored. Donor cells (9.4 ± 3.3%) were detected among MSCs. Thus, implanted MSCs remain localized at the site of administration and do not lose the ability to proliferate. These results suggest that MSCs could participate in the restoration of niches for donor hematopoietic cells or have an immunomodulatory effect, preventing repeated rejection of the graft. Perhaps, intraosseous implantation of MSCs contributes to the success of the second transplantation of hematopoietic stem cells and patient survival.

## 1. Introduction

Allogeneic transplantation of hematopoietic stem cells (allo-HSCT) remains the only method of treatment for many hematoblastosis patients.

Graft failure (GF) is a serious complication of allo-HSCT. With primary GF in the case of myeloablative conditioning (MAC), hematopoiesis of the patient is never restored and the only option is to repeat allo-HSCT. In the case of reduced intensity conditioning (RIC), there is a potential for patient hematopoiesis restoration [1]. The incidence of GF is 3.8–5.6%. Studies have suggested that GF is the result of the classical immune response of the recipient's immune cells (particularly the T cells), which remains after the pretransplant conditioning, to the donor antigens [1]. In addition, in GF patients in a prolonged aplasia, hematopoietic microenvironment could be fatally damaged, and their stromal cells fail to support hematopoiesis [2]. After allo-HSCT, the stromal microenvironment suffers from the effects of chemotherapeutic drugs [3, 4]. Not only does chemotherapy damage the bone marrow stroma but leukemia cells also adapt bone marrow niches to their own needs, altering the stromal microenvironment [4–6].

The structure of the stromal microenvironment includes mesenchymal stem cells, multipotent mesenchymal stromal cells (MSCs), colony-forming unit fibroblasts (CFU-F), and mature cells.

MSCs have been used since 2000 to treat various hematologic and autoimmune diseases, including acute graft versus host disease (aGvHD). Since 2008, in the National Research Center for Hematology, the study on the use of MSCs for aGvHD prevention has been conducted [7–9]. Since that time, for each patient, MSCs from bone marrow donors have been individually cultured. In this study, as in the vast majority of other studies using MSCs, cells were administered intravenously (IV). MSCs infused intravenously are firstly trapped in the lungs [10, 11]. It is not possible to detect donor MSCs in the body 7–10 days after IV injection [12, 13]. Hematopoietic cells enter the bone marrow according to the gradient of CXCL12 (SDF1) [14]. The CXCR4 receptor to this chemokine is weakly expressed on MSCs. Thus, MSCs introduced IV practically do not reach the bone marrow [15, 16].

When MSCs are IV cotransplanted at the time of allo-HSCT, donor MSCs are not found in the bone marrow stroma [13, 17]. Although, after the injection, MSCs may migrate into the inflammatory focus, studies have shown that their ability to migrate is small [18, 19]. In addition, when MSCs are used for bone and cartilage regeneration, the main problem is localizing MSCs at the appropriate site [20].

MSCs participate in the niches for hematopoietic stem cell formation and are thus elements that support hematopoiesis [21, 22]. Other stromal progenitor cells, such as colony-forming unit fibroblasts (CFU-Fs), as descendants of mesenchymal stem cells, participate in the formation of bone marrow stroma [23–25]. Both MSCs and CFU-Fs are able to proliferate and differentiate into bone, cartilage, and adipose tissues.

Donor MSCs in the case of GF can serve as a source of recovery for hematopoietic cell niches. During prolonged bone marrow aplasia, 3 patients intraosseously received MSCs from the bone marrow of hematopoietic cell donors. On average, 2 weeks after MSC administration, patients recovered their own hematopoiesis. Donor MSCs were found in recipient bone marrow three and five months following MSC implantation. Previous studies have shown that functionally adequate donor MSCs survive for a long time in the bone marrow of the patient [26]. In these cases, patients were not cotransplanted with hematopoietic stem cells from the donor. However, restoration of their own hematopoiesis suggested that MSCs perform a trophic function and contribute to the restoration of their own hematopoiesis. The results of this work became the basis for the combination of hematopoietic stem cell transplantation with intraosseous injection of MSCs derived from the bone marrow of hematopoietic cell donor.

In the Department of Bone Marrow Transplantation, 8 patients with GF after allogeneic bone marrow transplantation (allo-BMT) were observed. These patients received second allo-HSCTs from the same donor, and MSCs were injected intraosseously in an attempt to restore the stromal microenvironment. The long-term existence of donor MSCs in the bone marrow of these patients was shown. Six patients restored donor hematopoiesis.

## 2. Materials and Methods

### 2.1. Patients

Eight patients with GF after allo-BMT were observed in the Department of Bone Marrow Transplantation (Table 1). All patients received allo-BMT in complete remission. As GvHD prophylaxis patients received cyclosporine combined with methotrexate, some patients additionally received mycophenolate mofetil.

In all 8 cases, the first source of hematopoietic stem cells was bone marrow, whereas the second source of hematopoietic stem cells was peripheral blood stem cells from the same donor after mobilization via G-CSF. All patients received second allo-HSCTs and MSCs injected intraosseously (Table 2). At the first transplantation, no less than 2 × 10^8^ nucleated cells per kg of patient weight were injected, and at the second transplantation, no less than 3 × 10^6^ CD34 + cells per kg were injected. Except for patient 2, no GvHD cases occurred.

In the 2nd transplantation, MSCs were administered 1–6 hours before allo-HSCT under local anesthesia to the iliac crests on the right and left sides after receiving informed consent from the patient. Given that the MSCs for administration were obtained from several passages, the cells were thawed, washed from dimethylsulfoxide (DMSO) (Sigma-Aldrich, Steinheim, Germany), and suspended in 2 ml of 6% dextran (which has the trade name Poliglukin, produced by Public Corporation Biochimik, Russia). The cells were introduced into bone tissue in small aliquots of 100–200 *μ*l through 2 punctures of the skin and multiple punctures of the periosteum.

All works were conducted in accordance with the Declaration of Helsinki (1964). This study was approved by the local ethics committee, and the donors and patients provided written informed consent. A clinical trial (NCT03389919) has been ongoing at the National Research Center for Hematology since 2016.

### 2.2. MSCs

MSCs were derived from 25–30 ml of bone marrow received during donation for first transplantation from hematopoietic cell donors. For the separation of mononuclear cells, bone marrow was mixed with an equal volume of alpha-МЕМ media (HyClone, USA) containing 0.2% methylcellulose (1500 cP, Sigma-Aldrich, Steinheim, Germany). After 40 min, erythrocytes and granulocytes had mostly precipitated, while mononuclear cells remained in suspension. The upper fraction (suspension) was aspirated and centrifuged for 10 minutes at 450*g*. The sediment was suspended in a cultivation medium composed of alpha-MEM supplemented with 4% donor platelets [27], 2 mМ L-glutamine (HyClone, USA), 100 U/ml penicillin (Ferein, Russia) and 50 *μ*g/ml streptomycin (Ferein, Russia), and 2 *μ*/ml heparin (Sigma, USA). The cells were cultured at 27 × 10^6^ cells per T175 cm^2^ culture flask (Corning-Costar, USA). When a confluent monolayer of cells formed, the cells were washed with 0.02% EDTA (Sigma, USA) in a physiologic solution (Sigma-Aldrich, Steinheim, Germany) and were then trypsinized (MP Biomedicals, France). The cells were subsequently seeded at 4 × 10^3^ cells per cm^2^ of flask area. The cultures were maintained under hypoxic conditions at 37°C with a 5% CO_2_ and 5% O_2_ atmosphere. The MSCs were harvested in 6% dextran and cryopreserved with 10% DMSO.

The criteria for eligibility of the MSCs to be clinically applied included a spindle-shaped morphology, absence of visible clots, standard immunophenotype [28] for surface molecules [29], and proven ability to differentiate *in vitro* along osteogenic and adipogenic lineages [30]. MSCs were also examined for bacteria, viruses, and mycoplasma contamination before use as a routine standard procedure.

We performed bone marrow punctures for patients at 1-2, 4-5, and 9 months after simultaneous donor MSC implantation. We collected 2-3 ml of bone marrow from 1–4 independent punctures of the iliac spine for each patient. From the harvested bone marrow, MSCs were isolated and cultured according to the standard protocol described above, with the addition of 10% fetal calf serum (FCS) (Hyclone, Thermo Scientific, South Logan, USA) instead of plasma enriched with platelets. From these bone marrow samples, CFU-Fs were analyzed according to a standard protocol [9].

### 2.3. Chimerism Analysis

The proportion of chimeric DNA in patients' MSCs was determined at passages 1–3. MSCs from passage 0 were not included in the analysis due to the potential admixture of donor macrophages [31]. The immunophenotype of MSCs from passage 1 was determined. More than 99% of cells in each sample were CD90+/CD105+ and negative for hematopoietic cell markers (CD45, CD33, CD117, CD13, and CD34).

Cell DNA was isolated by the standard methods of extraction and salt precipitation with ethanol. The chimerism in MSCs was analyzed by the STR-PCR method (polymerase chain reaction with a panel of primers for the loci of short tandem repeats). The protocol uses reagents for multiplex analysis of 19 STR markers and the human amelogenin locus (CorDIS Plus C1000 Thermal Cycler). Fragment analysis was performed on a 3130 Genetic Analyzer. Data processing was conducted using GeneMapper v.4-0. The informative loci were preselected to monitor chimerisms in observed patients (Table 3). The percentage of donor chimerism was calculated using several standard formulas [32]. The ratio for minimal fluorescent signal to noise for minor DNA detection was 2 and higher. The ratio of base peak to noise was about 200. The sensitivity limit for chimerism investigation by means of STR-PCR with such conditions is about 1% in fragment analysis.

## 3. Results and Discussion

In all patients described, GF was observed (the number of WBCs at +30 days after allo-HSCT was 0.02–0.3 × 10^9^/L). Patients had pancytopenia and bone marrow aplasia. The majority of patients had mixed or their own hematopoiesis, and in some cases, molecular chimerism could not be determined due to the extremely scarce number of cells in the bone marrow sample. On the day of the second transplantation, performed 45–147 days after the first allo-BMT, the number of WBCs in peripheral blood of patients was 0.01 to 0.4 × 10^9^/L. Prior to the second transplantation of hematopoietic stem cells, all patients received intraosseous MSCs from the donor of hematopoietic stem cells. The attempts to infuse hematopoietic progenitor cells directly into the marrow, in the hope of accelerating engraftment, were performed 20 years ago [33]. A randomized trail comparing intraosseous and intravenous bone marrow transplantation revealed after 20-year follow-up that the procedure of intraosseous hematopoietic cell infusion is safe and the results are similar to those from the current clinical practice of intravenous infusion [34]. Recently, the safety of MSC intraosseous injection was demonstrated [35, 36]. These data allowed us to use intraosseous injection of mesenchymal stromal cells. No complications were observed in patients after intraosseous MSC implantation.

The reconstitution of donor hematopoiesis was detected in 6 out of 8 cases after intraosseous injection of MSCs. In 2 patients, there was no hematopoiesis recovery. One patient died of infectious complications within 76 days. The second patient is alive, however, with a WBC count of 0.01 × 10^9^/L. In the remaining 6 patients, the number of WBCs ranged from 1.3 to 3.1 × 10^9^/L at +30 days after the second transplantation.

Despite the temporary restoration of hematopoiesis, 2 patients died from infectious complications. In one patient (4F), donor leukocytes increased to 1.3 × 10^9^/L at +30 days after the second transplantation, but subsequently decreased to 0.5 × 10^9^/L due to infectious complications of mixed etiology (viral, bacterial, and fungal), and the patient died 63 days after the 2nd transplantation. In the second patient (2P), one month after the transplantation, the WBC count was 1.5 × 10^9^/L, and after 4 days, gastrointestinal acute GVHD grade II was diagnosed, and immunosuppressive therapy was started. The number of WBCs decreased to 0.1 × 10^9^/L. The patient died from infectious complications 68 days after the 2nd transplantation.

The remaining patients are alive and retain donor hematopoiesis.

In all patients with donor hematopoiesis, at the time of the diagnostic punctures, bone marrow was harvested from 1–4 sites near the location of MSC injection to analyze chimerisms in stromal progenitor cells. CFU-Fs and MSCs were developed from the bone marrow of these patients. In 2 patients without a hematopoietic restoration due to a low bone marrow cell count (only 200,000–300,000 cells were available for cultivation), cultures of MSCs and CFU-Fs could not be obtained.

**Figure 1:**
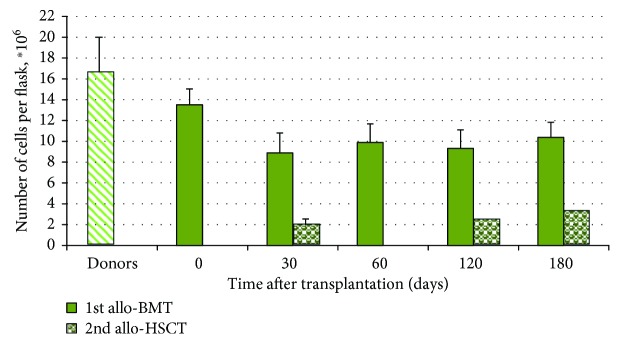
Cumulative MSC production for 3 passages in patients after allogeneic hematopoietic stem cell transplantation. The data are presented as the mean ± standard error (M ± SE), donors—88 MSC samples; patients before allo-BMT (point 0)—44 samples; patients after 1st allo-BMT—35 samples; patients after 2nd allo-HSCT—5 samples for 30 days and 2 samples for 120 and 180 days.

The total cell production of MSCs for 3 passages in the studied patients was significantly reduced during the entire observation period compared to that of donors and patients after a single allogeneic transplantation (Figure 1). The data from patients with hematological malignancies after allo-HSCT [3] and average values of cumulative MSC production in the studied patients were compared.

In patients after therapy before transplantation, the total cell production of MSCs decreased compared to that in healthy donors. After the 1st transplantation, the MSC cell production significantly decreased 1.5 times more and remained reduced at all the studied points. It was impossible to study MSCs in patients with GF before the 2nd transplantation because of the extremely poor cellular composition of the bone marrow. After the 2nd transplantation, the MSC cell production was reduced 3–5 times compared to that after the 1st. It is unlikely that conditioning with fludarabine and melphalan had such a strong damage effect. Most likely, this was a consequence of fatal damage to stromal progenitors even before the 2nd transplant, which could be the cause of GF. In 5 patients with restoration of donor hematopoiesis, donor stromal cells were detected among MSCs and CFU-Fs at 1–9 months after the second transplantation. In all patients, more than 99% of MSCs at passage 1 were CD90+/CD105+, and cells positive for CD45, CD33, CD117, CD13, and CD34 were not detected. The proportion of donor cells varied in MSCs cultured from bone marrow that was simultaneously aspirated from different sites of the iliac crest. The data on chimerisms in bone marrow, MSCs, and CFU-Fs (the mean values ± standard error) are presented in Table 4.

In bone marrow samples from patients, the proportion of donor CFU-Fs was significantly higher (65.5 ± 4.5%) than the proportion of MSCs, suggesting that stromal progenitor cells (CFU-F)—the descendants of MSCs of donor origin—predominate and are more functionally active than the own progenitor cells of the recipient.

It has long been suggested that the main cause of GF is rejection of the transplant by the host cytotoxic T-cells [37, 38]. There is evidence that the recipient's stromal cells are attacked by donor lymphocytes and lose the ability to support hematopoiesis [39, 40]. In GvHD, niches for stem cells in the bone marrow may be damaged [41]. It is proven that stromal progenitors are not transferred by intravenous administration [13]; however, when implanted under the skin or the kidney capsule, these cells form hematopoietic ectopic foci, whose stromal microenvironment consists of donor cells and hematopoietic cells belonging to the recipient [42–44].

Repeated allo-HSCT with the simultaneous introduction of donor MSCs intraosseously to patients with GF was performed in the Department of Bone Marrow Transplantation based on the available data on the potential damage of the stromal microenvironment and detection of donor MSCs in the bone marrow a few months after implantation into bone tissue [26]. In 6 out of 8 patients who received this MSC implantation, donor hematopoiesis was restored. Notably, the site of bone marrow collection in patients and site of donor MSC injection only approximately coincided. In this regard, all of the data on chimerisms are relative and were confirmed by the significant variation of the chimerism of MSCs observed after the simultaneous sampling of the bone marrow from several points. The detection of donor MSCs in the bone marrow of patients long after implantation shows that these cells performed not only a trophic function but also shared and participated in the construction of the hematopoietic microenvironment, supporting donor hematopoiesis. On average, only 245 ± 33 × 10^6^ MSCs were implanted in each patient. Compared with the entire hematopoietic area, where approximately 5% of stromal cells are contained, this dose is an insignificant amount. In 2 patients who did not recover hematopoiesis, MSCs could not be obtained because of low bone marrow cellularity. In patients with restored hematopoiesis, MSCs isolated from their bone marrow at 1–9 months after the second transplantation proliferated at least during 3 passages in culture.

The total cell production of MSCs in these patients was significantly lower than that in donors (6.8 ± 1 times) and patients after a single bone marrow transplantation with normal restoration of donor hematopoiesis (3.8 ± 0.4 times) [4]. Administration of a small dose of donor MSCs was sufficient to restore donor hematopoiesis in these patients. On average, 9.4 ± 3.3% of donor MSCs were observed in all bone marrow samples from the patients at all time points.

## 4. Conclusions

Thus, donor MSCs might improve the stromal microenvironment of patients and participate in the recovery of donor hematopoiesis. The results obtained in the present study suggest the possibility to use of an intraosseous injection of MSCs from bone marrow donors for the treatment of patients with GF.

## Figures and Tables

**Table 1:** Characteristics of the 1st transplantation.

Patient	Diagnosis	Gender/age	Donor	Conditioning regimen	Time to 2nd transplantation, days	Leucocytes, ×10^9^/L at the time of the 2nd transplantation	Graft rejection
1 M	AML	m/23	Unrelated matched	MAC	50	0.01	Primary
2 P	MDS	f/56	Related matched	RIC	108	0.4	Primary
3 CH	MDS	f/40	Unrelated mismatched	RIC	83	0.04	Primary
4 F	AML	f/42	Unrelated matched	RIC	147	0.01	Secondary
5 IV	CMML	m/30	Unrelated matched	MAC	64	0.01	Primary
6 BU	AML	m/32	Unrelated mismatched	MAC	50	0.02	Primary
7 SA	AML	f/53	Unrelated mismatched	RIC	55	0.05	Primary
8 CHA	AML	f/32	Unrelated mismatched	MAC	45	0.02	Primary

AML: acute myeloid leukemia; MDS: myelodysplastic syndrome; CMML: chronic myelomonocytic leukemia; RIC: reduced intensity conditioning (fludarabine phosphate (30 mg/m^2^/day for 6 days) combined with busulfan 4 mg/kg/day for 2 days and antithymocitic globulin 10 mg/kg/day for 4 days); MAC: myeloablative conditioning (cyclophosphamide 60 mg/kg/day for 2 days combined with busulfan 4 mg/kg/day for 4 days).

**Table 2:** Characteristics of the 2nd transplantation.

Patient	2nd conditioning regimen	MSC dose, ^∗^10^6^ cells	MSC dose, ^∗^10^6^ cells/per Kg of body weight	WBC count recovery, leucocytes ^∗^10^9^ (+30 days)	Day of PLT count recovery, 50^∗^10^9^	Death, days after the 2nd transplantation	Cause of death
1 M	RIC	369	5.4	1.3	96		
2 P	Melf	189	2.8	1.5	42	68	GvHD, infection
3 CH	Melf + Fludara	187	3.5	1.6	33		
4 F	Melf + Fludara	194	2.4	1.3	49	63	Infection
5 IV	Melf + Fludara	373	5.3	0.02	No	76	Infection
6 BU	Melf + Fludara	174	2.1	0.01	No		
7 SA	Melf + Fludara	230	3.8	3.1	127		
8 CHA	Melf + Fludara	162	3.1	2.2	33		

**Table 3:** The informative loci for chimerism analysis.

Patient	Informative loci
1 M	D5S818; CSF1PO; D13S317
2 P	D12S391; D13S317; CSF1PO; D18S51
3 CH	D12S391; D1S1656; D10S1248; FGA; D13S317; D8S1179
4 F	FGA; D8S1179
7 SA	Amelogenin Y, TH01, D13S317, D5S818, SE33
8 CHA	D10S1248, FGA, D18S51, D8S1179

**Table 4:** Proportion of donor cells in the bone marrow (BM), CFU-Fs, and MSCs.

Proportion of donor chimerism, %									
Time after MSC injection directly into the trabecular bone area									
Patients	1-2 months			4-5 months			9 months		
	BM	CFU-F	MSCs	BM	CFU-F	MSCs	BM	CFU-F	MSCs
1 M	25–50	ND	6.9 ± 0.5	100	78 ± 4	3.2 ± 2.2	100	62 ± 5	7.2 ± 0.6
2 P	100	ND	5.0 ± 0.9	Death on the 68th day					
3 CH	100	65.5 ± 19.7	2.0 ± 0.8	100	57 ± 6	21.7 ± 2.0	100	100	7.3 ± 2.4
4 F	100	ND	3.1 ± 0.002	Death on the 63rd day					
7 SA	100	79.5 ± 12.1	22.3 ± 4.6	ND	ND	ND	ND	ND	ND
8 CHA	100	50.0 ± 2.0	9.3 ± 4.2	ND	ND	ND	ND	ND	ND

For each patient, MSCs were obtained from the bone marrow taken from 2–4 points of the iliac bones. The data are presented as the average percentage of donor MSCs at 1–3 passages from all investigated bone marrow locations ± standard error. ND: no data. The sensitivity limit of method used was 1%.
